# Amyloid-like aggregation of provasopressin in diabetes insipidus and secretory granule sorting

**DOI:** 10.1186/s12915-017-0347-9

**Published:** 2017-01-26

**Authors:** Nicole Beuret, Franziska Hasler, Cristina Prescianotto-Baschong, Julia Birk, Jonas Rutishauser, Martin Spiess

**Affiliations:** Biozentrum, University of Basel, Klingelbergstrasse 70, CH-4056 Basel, Switzerland

**Keywords:** Amyloid aggregation, Regulated secretion, Secretory granules, Vasopressin

## Abstract

**Background:**

Aggregation of peptide hormone precursors in the trans-Golgi network is an essential process in the biogenesis of secretory granules in endocrine cells. It has recently been proposed that this aggregation corresponds to the formation of functional amyloids. Our previous finding that dominant mutations in provasopressin, which cause cell degeneration and diabetes insipidus, prevent native folding and produce fibrillar aggregates in the endoplasmic reticulum (ER) might thus reflect mislocalized amyloid formation by sequences that evolved to mediate granule sorting.

**Results:**

Here we identified two sequences responsible for fibrillar aggregation of mutant precursors in the ER: the N-terminal vasopressin nonapeptide and the C-terminal glycopeptide. To test their role in granule sorting, the glycopeptide was deleted and/or vasopressin mutated to inactivate ER aggregation while still permitting precursor folding and ER exit. These mutations strongly reduced sorting into granules and regulated secretion in endocrine AtT20 cells.

**Conclusion:**

The same sequences — vasopressin and the glycopeptide — mediate physiological aggregation of the wild-type hormone precursor into secretory granules and the pathological fibrillar aggregation of disease mutants in the ER. These findings support the amyloid hypothesis for secretory granule biogenesis.

**Electronic supplementary material:**

The online version of this article (doi:10.1186/s12915-017-0347-9) contains supplementary material, which is available to authorized users.

## Background

**Fig. 1:**
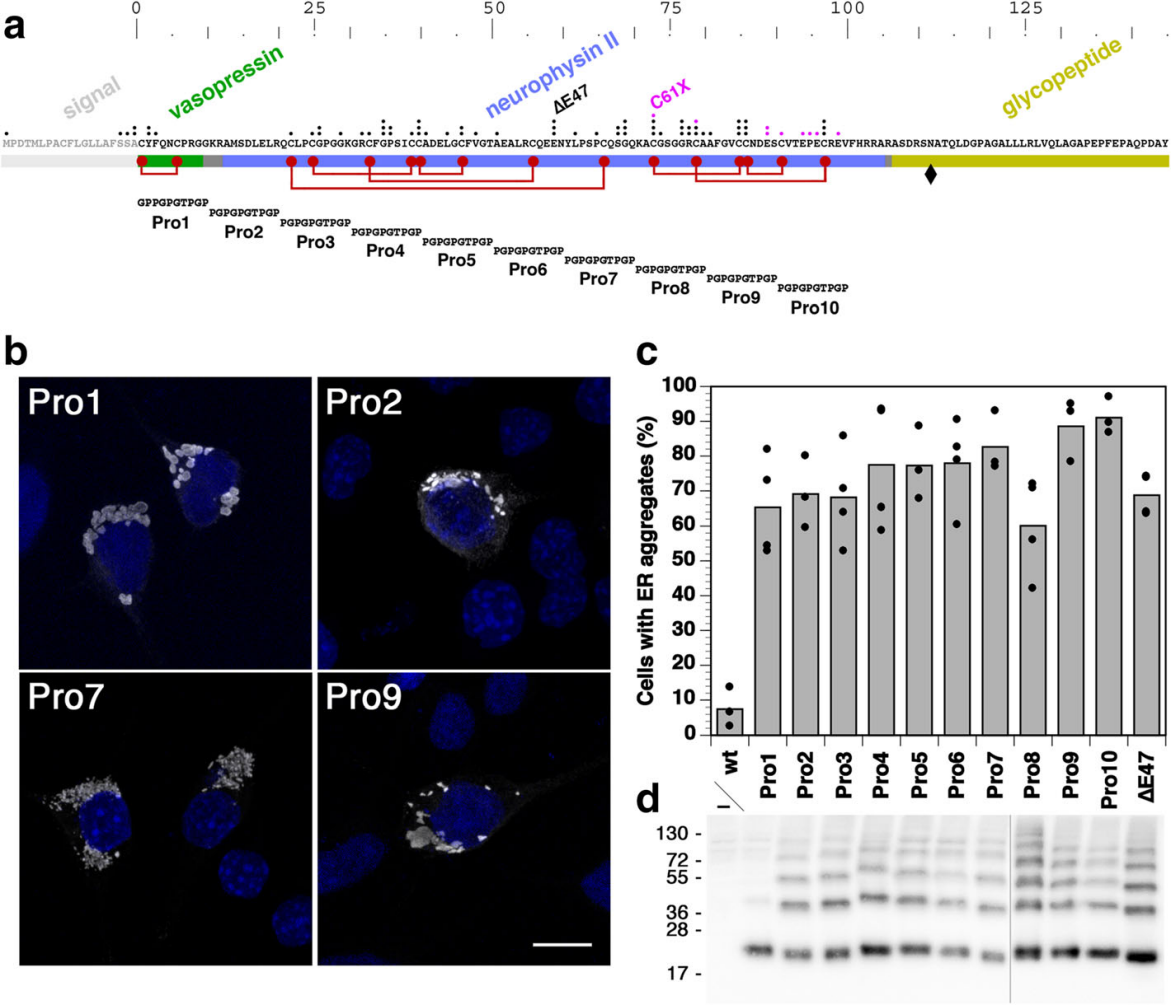
Pro/Gly scan through full-length provasopressin suggests more than one ER aggregating segment. **a** Domain organization of preprovasopressin. Cysteines are indicated by *red dots*, disulfide bonds by *red lines*, and glycosylation by a *black diamond. Dots* above the sequence indicate distinct mutations causing autosomal dominant neurohypophyseal DI (missense or deletion in *black*, nonsense or frameshift in *pink*). The natural mutants used in this study, ∆E47 and C61X, are labeled. The scale indicates the number of the amino acids in provasopressin. In constructs Pro1–Pro10 successive segments of 10 residues in provasopressin were replaced by proline/glycine-rich sequences as illustrated. **b** The constructs were expressed in HN10 cells for 2 days and analyzed by immunofluorescence staining. Pro1/2/7/9-expressing cells are shown as examples. Nuclei were stained with DAPI (*blue*). *Bar*: 10 μm. **c** The fraction of expressing cells with ER aggregates was quantified and plotted (mean and individual values of three or four independent transfections (as indicated), analyzing ~200 expressing cells per transfection as described in “Methods”). **d** Immunoblot analysis of transfected cells after separation by reducing sodium dodecyl sulfate (*SDS*)-gel electrophoresis is shown. Considerable amounts of SDS- and dithiothreitol-resistant provasopressin oligomers were detected for all proteins except Pro1. Molecular weight standards are indicated in kilodaltons

The peptide hormone vasopressin regulates water homeostasis by binding to receptors of the renal collecting duct to promote increased water reabsorption from the urine. Absence of vasopressin causes diabetes insipidus (DI), a disease characterized by loss of large amounts of unconcentrated urine and increased thirst. The hormone is synthesized by vasopressinergic neurons in the hypothalamus as part of a precursor protein (provasopressin; illustrated in Fig. 1a) composed of a cleavable signal for endoplasmic reticulum (ER) targeting, the nonapeptide hormone sequence, the 93-amino acid neurophysin II (NPII) domain, and a glycopeptide of 39 residues of unknown function. The folded precursor is stabilized by seven disulfide bonds in NPII and one in the vasopressin sequence. Crystal structures show vasopressin to fold with its N-terminus into a binding pocket of NPII [1], which is essential for NPII folding and export of the precursor from the ER [2].

Folded provasopressin is transported through the Golgi to the trans-Golgi network (TGN) and sorted into dense-core secretory granules, where it is cleaved by prohormone convertases into its three constituents and where the 10-amino acid vasopressin sequence CYFQNCPRGG is converted to the C-terminally amidated nonapeptide hormone CYFQNCPRG-NH_2_. The granules are transported to nerve terminals in the posterior pituitary for regulated release. While the detailed mechanism of proprotein sorting into granules is not established, it is clear that self-aggregation of regulated cargo at the TGN contributes to granule formation [3–6]. Indeed, expression of provasopressin (or of other regulated cargo proteins) was shown to be sufficient to produce dense granule-like structures in fibroblasts and other cells lacking endocrine-specific machinery [7].

More than 70 different mutations causing familial DI have been identified throughout the precursor sequence, except the glycopeptide. Most strikingly, all but one of them are dominant, causing what is known as autosomal dominant neurohypophyseal DI (ADNDI). Patients who are heterozygous lack vasopressin and develop DI gradually within months or years after birth. Postmortem biopsies revealed the loss of vasopressinergic neurons (see, e.g., [8–10]), suggesting that mutant provasopressin causes their degeneration. This phenotype was reproduced in knock-in mouse models, where expression of the human DI mutant C67X (codon for cysteine 67 of NPII mutated to a stop codon) led to progressive loss of vasopressinergic neurons [11, 12]. In transfected cell lines, mutant proteins were found to be retained in the ER [13] and to some extent degraded via cytoplasmic proteasomes [14], as expected for polypeptides that are unable to fold. Most interestingly, ADNDI mutant precursors were found to form fibrillar aggregates in the ER of transfected cells and the purified mutant protein was shown to form fibers in vitro [15]. These results suggested that ADNDI belongs to the group of neurodegenerative diseases associated with fibrillar protein aggregation, similar to the amyloid diseases, with the distinction that aggregation does not occur in the cytosol or extracellularly, but in the ER lumen. Analysis in mice showed that aggregates produced by mutant precursors induce autophagy-associated cell death [12].

Provasopressin thus contains sequences with the potential to form pathological aggregates when mutations interfere with native folding of the precursor. Such sequences may in fact have a physiological role. In a recent study, it was proposed that aggregation of peptide hormones into secretory granules corresponds to the formation of functional amyloids [16]. This was mainly based on the observation that secretory granules of the pituitary — including those containing vasopressin — stained positive for thioflavin S, and that many mature peptide hormones — including vasopressin — could form fibrils in vitro. In this light, the formation of pathological aggregates in the ER of folding-deficient provasopressin may be a consequence of a physiological aggregation motif that evolved for the formation of functional amyloids in the TGN. To test this hypothesis, it is important to identify the sequence motifs responsible for both pathological aggregation in the ER and physiological aggregation of the natively folded precursor into granules at the TGN. Previous analyses did not allow a clear conclusion on the sequences required for granule sorting. Using green fluorescent protein (GFP) fusion constructs, residues 1–27 of provasopressin, but not 1–10 (the hormone sequence) or 13–27 (the N-terminus of NPII) alone, were reported to mediate granule localization in one study [17] and the NPII domain in another [18]. In general, identification of signals for sorting or aggregation into the secretory granules has not been very successful, to a large extent because mutagenesis of precursor proteins frequently results in misfolded products retained in the ER.

Here we first identified by scanning mutagenesis and deletion analysis two independent segments responsible for ER aggregation: the nonapeptide hormone sequence and the glycopeptide. Analysis of folding-competent precursor proteins mutated in these segments indicates that the same sequences also mediate granule aggregation and sorting. These findings thus support the hypothesis that, in ADNDI, sequences that have evolved to physiologically aggregate the folded protein at the TGN cause premature aggregation of folding-incompetent mutant proteins in the ER with toxic effects to the cell.

## Results

To analyze the aggregation properties of wild-type and mutant vasopressin precursors, we expressed them in HN10 neuroblastoma cells, which were cultured in differentiation medium to induce the formation of neuronal processes. By immunofluorescence microscopy, wild-type provasopressin was detected in secretory granules as fine puncta located mostly in the cellular processes and localizing with the granule marker chromogranin A (CgA; Additional file 1: Figure S1, filled arrowheads). In contrast, the natural ADNDI mutant ∆E47 accumulated in most of the cells in larger aggregations within the cell body which were costained with antibodies directed against the KDEL ER retention motif (open arrowheads), in agreement with our previous observation of ER aggregations in COS and Neuro2A cells [15].

### Provasopressin contains more than one aggregating sequence

To identify the sequence motif(s) necessary to form fibrillar aggregates of misfolded provasopressin, mutant constructs Pro1–Pro10 were produced with a 10-residue long proline-/glycine-rich sequence scanning through the first 100 amino acids of provasopressin (vasopressin and NPII), as illustrated in Fig. 1a. Prolines and glycines disrupt secondary structures, particularly β-sheets, the essential element of amyloids. The sequence PGPGPGTPGP was used to replace each segment of 10 amino acids in the vasopressin and NPII portions of the full-length protein. For Pro1 the first two residues were swapped (GPPGPGTPGP) to allow signal cleavage, which is prevented by proline at the cleavage site. All 10 constructs also disrupt precursor folding.

Surprisingly, all Pro/Gly mutant provasopressins were found to aggregate efficiently in the ER of expressing HN10 cells (Fig. 1b and c). No single 10-amino acid segment of vasopressin-NPII is thus necessary for ER aggregation, and there must be more than one segment in provasopressin sufficient to cause aggregation. When transfected cells were lysed without reduction and analyzed by immunoblotting after nonreducing sodium dodecyl sulfate (SDS)-gel electrophoresis, the accumulated intracellular mutant proteins did not enter the separating gel, suggesting that they aggregated in a highly disulfide-linked form. Upon addition of 100 mM dithiothreitol (DTT) and boiling, they entered the separating gel, but all mutant constructs except Pro1 were detected to a large extent as SDS/DTT-resistant homo-oligomers, typical of some amyloid aggregates (Fig. 1d).

### The shortest known pathogenic provasopressin mutant forms fibrillar aggregates in the ER

**Fig. 2:**
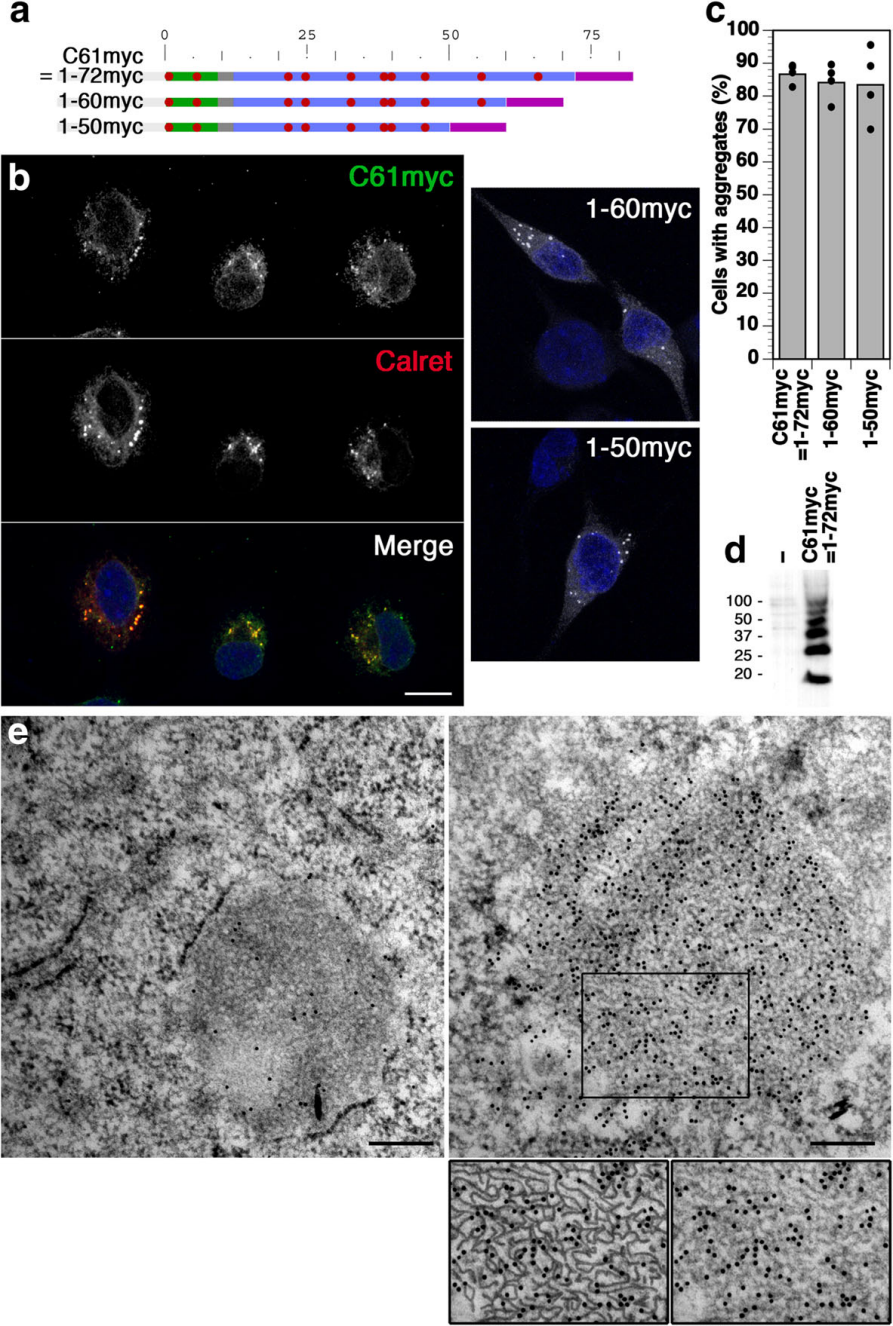
ER aggregation of C-terminally truncated forms of provasopressin. **a** Schematic representation of C-terminally truncated provasopressin mutants C61myc (corresponding to 1-72myc), 1-60myc, and 1-50myc. The myc epitope is shown in *purple*. **b** These constructs were expressed in HN10 cells and visualized by immunofluorescence microscopy. Cells expressing C61myc were costained for the ER chaperone calreticulin. Nuclei were stained with DAPI (*blue*). *Bar*: 10 μm. **c** The fraction of expressing cells with ER aggregates was quantified and plotted (mean and individual values of four independent transfections, analyzing ~200 expressing cells per transfection). **d** Immunoblot analysis of untransfected cells (*−*) and transfected cells expressing C61myc after reducing SDS-gel electrophoresis. Molecular weight standards are indicated in kilodaltons. **e** Electron micrographs of C61myc aggregates forming a fibrillar network decorated with 10-nm gold. The surrounding rough ER membrane is visible in the *left micrograph. Bars*: 200 nm. *Below*, a section is shown enlarged with and without fibrillar lines highlighted for clarity

To reduce the complexity of the system, we focused on the shortest known pathogenic ADNDI mutant, C61X, in which the codon for Cys-61 in NPII is altered to a stop codon. The protein thus corresponds to the N-terminal half (residues 1–72) of the precursor. Since our antibody does not recognize this part of the protein, a myc-tagged version of C61X, C61myc (Fig. 2a), was expressed in HN10 cells and found to produce aggregates positive for the ER chaperone calreticulin in the majority of expressing cells (Fig. 2b and c) as well as SDS/DTT-resistant oligomers (Fig. 2d). By electron microscopy (Fig. 2e), these aggregates, as identified by immunogold labeling, appeared to be made up of a network of fibrils similar to those formed by full-size mutant provasopressin [15]. At least one aggregate-forming element thus resides within the N-terminal 72 residues of provasopressin. In addition, the correlation between aggregation and pathogenicity is supported by this shortest DI mutant.

Provasopressin deletion constructs further truncated to the N-terminal 60 or 50 residues and fused to a myc epitope still produced large aggregates detectable by immunofluorescence (Fig. 2b and c), indicating that an aggregating sequence is located within the N-terminal 50 residues. A further truncation mutant, 1-40myc, could not be detected by immunofluorescence microscopy of transfected cells, most likely because the protein was too short for cotranslational translocation and was rapidly degraded in the cytosol.

### The vasopressin segment is necessary for aggregation of provasopressin 1–75 and sufficient for ER aggregation of a reporter protein

**Fig. 3:**
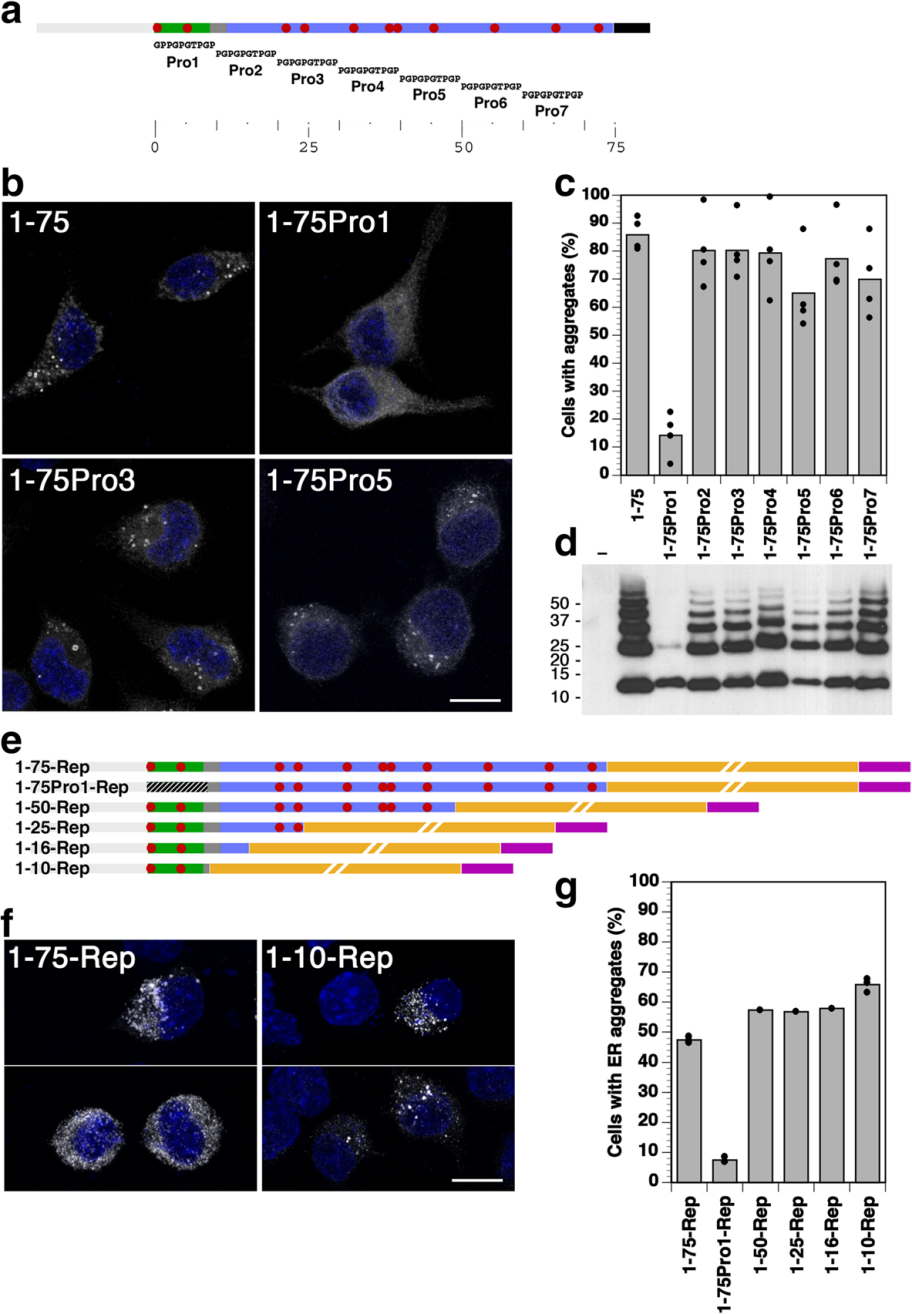
The vasopressin nonapeptide is necessary for ER aggregation of provasopressin 1–75 and sufficient to aggregate a reporter construct. **a** Schematic presentation of provasopressin 1–75 (with a C-terminal His_6_ tag in *black*) and its proline/glycine scanning mutants 1–75Pro1–7. **b** Immunofluorescence localization of example constructs expressed in HN10 cells. The indicated constructs fused to a His_6_ tag were transfected into HN10 cells and fluorescently stained using an anti-His_6_ antibody. *Bar*: 10 μm. **c** Aggregation frequency of these constructs was quantified as before (mean and individual values of four independent transfections, analyzing ~200 expressing cells per transfection). **d** Transfected cells were lysed, separated by reducing SDS-gel electrophoresis, and subjected to immunoblot analysis to detect intracellularly accumulated provasopressin constructs. Molecular weight standards are indicated in kilodaltons. **e** Truncated provasopressin sequences 1–75 (positive control), 1–75Pro1 (negative control), 1–50, 1–25, 1–16, and 1–10 were fused to a reporter sequence (*Rep*) consisting of the C-terminal fragment 101–218 of glutathione S-transferase (*GST*, *orange*) with a myc epitope (in *purple*). **f** Immunofluorescence localization of indicated constructs expressed in HN10 cells. *Bar*: 10 μm. **g** Aggregation frequency of the constructs shown in panel **e** was quantified as before (mean and individual values of three independent transfections for 1–75-Rep, 1–75Pro1-Rep, and 1–10-Rep, and single determinations for the others, analyzing ~100 expressing cells per transfection)

The Pro/Gly scan was now repeated for the N-terminal half of provasopressin (residues 1–75) alone, as illustrated in Fig. 3a. Of the seven constructs, only 1–75Pro1 did not produce ER aggregates in the vast majority of expressing cells (Fig. 3b and c). Again, the Pro1 mutation was the only one not generating SDS- and DTT-resistant homo-oligomers (Fig. 3d). These results show that a single 10-amino acid segment at the very N-terminus of the protein is necessary for aggregation. This segment corresponds to the vasopressin hormone sequence.

To define the sequence sufficient for aggregation, N-terminal segments of preprovasopressin were fused to a reporter polypeptide (Rep) consisting of a fragment of glutathione S-transferase (GST; residues 101–218) that cannot fold and is thus retained in the ER. Construct 1–75-Rep, in which the signal sequence and residues 1–75 of provasopressin were fused to the reporter, aggregated in approximately 50% of expressing HN10 cells, i.e., somewhat less than 1–75 alone. As expected, 1–75Pro1-Rep did not significantly aggregate at all (Fig. 3e–g). C-terminally truncated provasopressin segments corresponding to the N-terminal 50, 25, 16, and 10 residues all still produced intracellular aggregates. The vasopressin sequence CYFQNCPRGG is thus not only necessary, but also sufficient to cause aggregation of a polypeptide.

### The glycopeptide mediates fibrillar aggregation of provasopressin in the absence of the hormone sequence

**Fig. 4:**
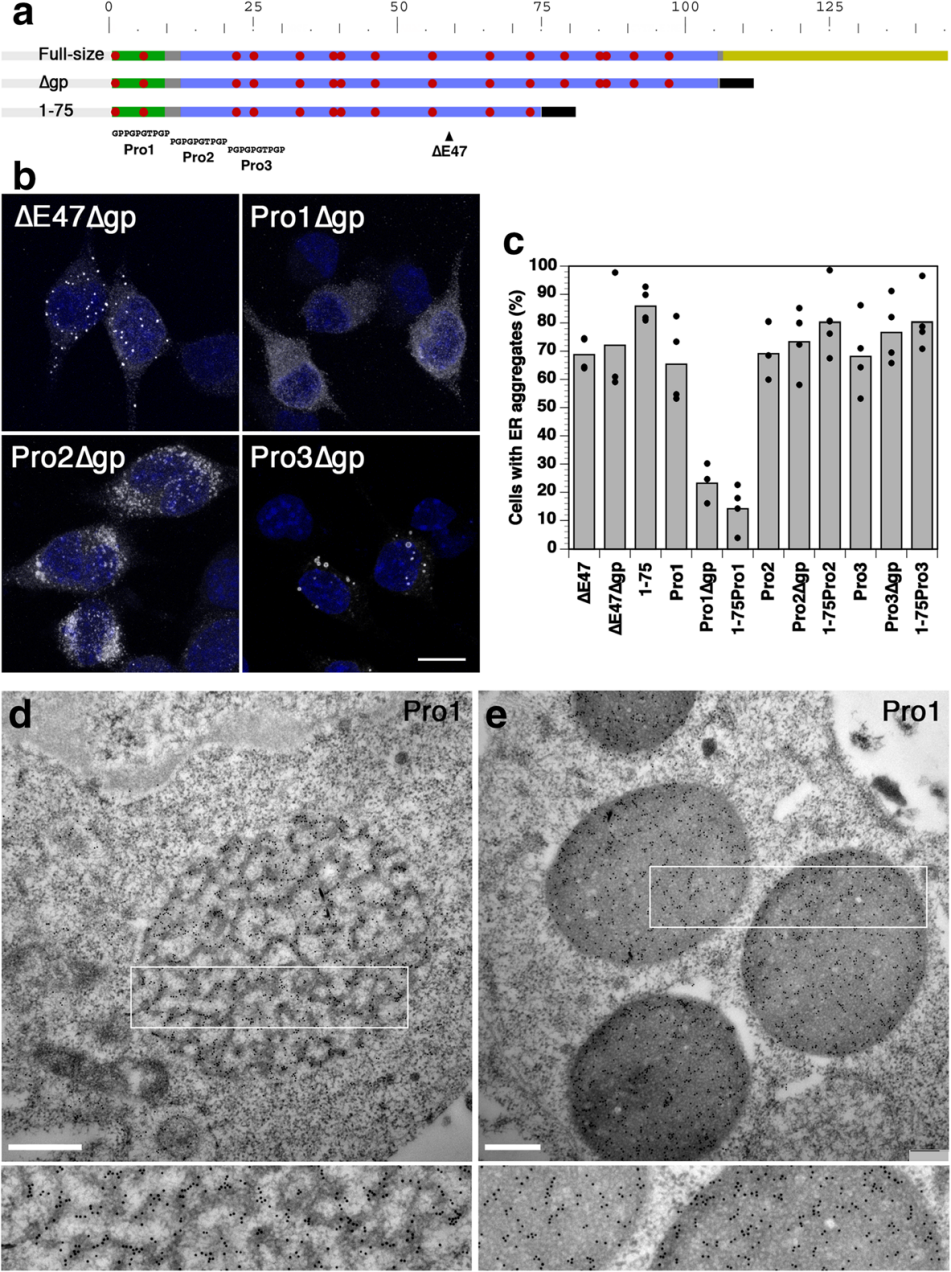
The glycopeptide sequence contains a second aggregation sequence. **a** Full-size provasopressin and deletion mutants lacking the glycopeptide (∆*gp*) or the C-terminal half of the protein (1–75), with or without the ∆E47 point mutation or the Pro1, Pro2, or Pro3 replacement (as indicated) were constructed to localize the second aggregation sequence. The truncated proteins were provided with a His_6_ tag (*black bar*). **b** Constructs were expressed in HN10 cells and analyzed by immunofluorescence as shown for selected examples. *Bar*: 10 μm. **c** The fraction of expressing cells forming ER aggregates was quantified as before (mean and individual values of three or four independent transfections (as indicated), analyzing ~200 expressing cells per transfection). For comparison, values for Pro1/2/3, 1–75, and 1–75Pro1/2/3 from Figs. 1c and 3c, respectively, are also shown. **d**, **e** Cells expressing construct Pro1, which lacks the N-terminal aggregation sequence, were analyzed by electron microscopy with immunogold labeling. Aggregates formed via the glycopeptide sequence showed different degrees of compaction (see also Additional file 2: Figure S2). Some aggregates contained dense and light regions (**d**), while others were almost completely dense (**e**). *Bars*: 500 nm. *Enlargements of the boxed regions below* reveal a fibrillar network within the aggregates

**Fig. 5:**
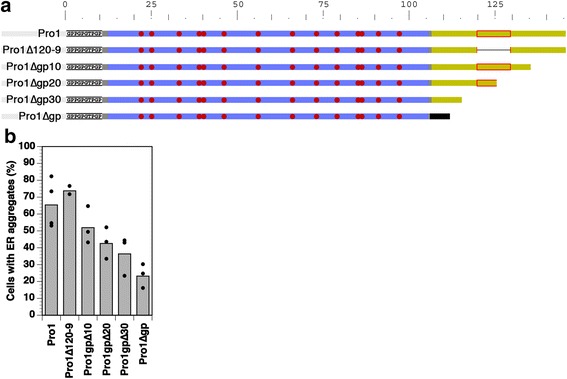
Deletion analysis of the glycopeptide with respect to ER aggregation. **a** Several amyloid prediction programs (including Aggrescan [42], Amylpred [43], and FoldAmyloid [44]) pointed to the sequence 120–129 in the middle of the glycopeptide sequence as potentially amyloidogenic. In construct Pro1∆120-9, this sequence was deleted. In addition, the glycopeptide was gradually truncated from the C-terminus in Pro1∆gp10/20/30. **b** Formation of aggregates in transfected HN10 cells revealed no significant effect of deleting the central sequence, but a gradual reduction of aggregate formation upon C-terminal truncation. The mean and individual values of two to four independent transfections (as indicated), analyzing ~200 expressing cells per transfection, are shown

To localize the additional aggregating sequence(s) in provasopressin, the first three Pro/Gly mutations (Pro1/2/3) were analyzed in the context of the full-size protein, of a truncated version lacking the C-terminal glycopeptide segment (∆gp), and of the N-terminal 75 residues (1–75, fused to a His_6_ tag; Fig. 4a). Deletion of the glycopetide dramatically reduced aggregation in the context of the Pro1 construct, but not Pro2 or Pro3 (Fig. 4b and c). The glycopeptide thus contains an independent aggregating activity. Gradual C-terminal deletion of the glycopeptide resulted in a progressive reduction of ER aggregation (Fig. 5).

When analyzed by electron microscopy, provasopressin mutants Pro1 and Pro1∆E47 lacking the hormone sequence, but containing the glycopeptide, were found in aggregations made up of a network of fibers (Fig. 4d and e, and Additional file 2: Figure S2). In some aggregate structures, the network formed regions of higher and lower density, which were also decorated by immunogold labeling to a larger and smaller extent, respectively (Fig. 4d and Additional file 2: Figure S2A and C). In other cases, the dense regions appeared to coalesce or even fill the structures entirely (Fig. 4e and Additional file 2: Figure S2B and D).

### A vasopressin mutant deficient in ER aggregation but permissive of precursor folding

**Fig. 6:**
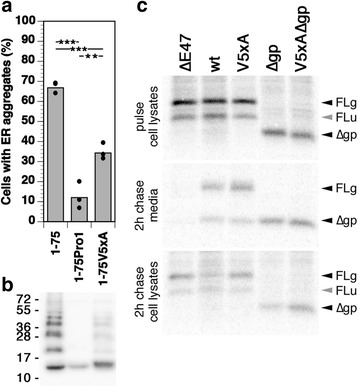
V5xA mutation reduces ER aggregation while still permitting precursor folding and secretion. **a** The fraction of cells expressing 1–75, 1–75Pro1, or 1–75V5xA forming ER aggregates was quantified as before (mean and individual values of three independent transfections, analyzing ~100 expressing cells per transfection). All pairwise comparisons are significantly different according to an unpaired two-tailed *t* test (**p < 0.01; ***p < 0.001). **b** Transfected cells were lysed, separated by reducing SDS-gel electrophoresis, and subjected to immunoblot analysis to detect intracellularly accumulated provasopressin constructs. Molecular weight standards are indicated (in kilodaltons). **c** Transfected HN10 cells expressing the indicated provasopressin constructs were labeled with [^35^S]methionine for 30 min and chased for an additional 2 h. From cell lysates and media, products were immunoprecipitated and analyzed by gel electrophoresis and autoradiography. The positions of full-size glycosylated (*FLg*) and unglycosylated forms (*FLu*) and of constructs without glycopeptide (∆*gp*) are indicated

To test the role of the glycopeptide in granule sorting, it can simply be deleted, since this segment is not required for vasopressin–NPII folding and ER exit. To test the contribution of the vasopressin segment in granule sorting is more difficult, since its interaction with NPII is required for folding. We aimed at generating a mutant vasopressin sequence that has lost its ability to aggregate in the ER, but still allows precursor folding and ER exit, and thus can be tested for granule sorting. The crystal structure of vasopressin bound to NPII (1JK4 [1]) shows that the N-terminus, C1 and C6 with their disulfide bond, as well as Y2 are buried inside NPII (illustrated in Additional file 3: Figure S3) and thus are essential for precursor folding. The side chains of F3, Q4, N5, P7, and R8 appear to be exposed to the solvent. We therefore mutated the vasopressin sequence CYFQNCPRG to CYAAACAAG in the context of the full-size precursor (V5xA), of a construct lacking the glycopeptide (V5xA∆gp), and as a truncated and thus folding-incompetent mutant (1–75V5xA). Expression of 1–75V5xA produced significantly fewer cells with aggregates than construct 1–75 with the wild-type vasopressin sequence, although more than 1–75Pro1 (Fig. 6a). The V5xA mutations thus strongly reduce the proteins’ ability to aggregate in the ER, but do not completely eliminate it. Similarly, the amount of SDS- and DTT-resistant cellular oligomers was clearly reduced by the 5xA mutations (to ~35%, compared to ~75% for 1–75 and a background level ~20% for 1–75Pro1), but again not entirely abolished (Fig. 6b).

To assess whether the fivefold alanine mutation allows precursor folding, constructs V5xA and V5xA∆gp were expressed in HN10 cells in parallel with the corresponding proteins with wild-type vasopressin and with the folding-deficient mutant ∆E47. Upon 30 min of pulse labeling with [^35^S]methionine/cysteine (Fig. 6c), the full-length constructs were produced mostly as glycosylated and to a lesser extent as unglycosylated protein (as observed previously [15]). During a subsequent 2-h chase, ∆E47 was entirely retained in the cell. In contrast, all other mutant proteins, except the unglycosylated full-length products, were secreted with similar efficiency as wild-type provasopressin, indicating that the V5xA mutant vasopressin to a significant extent was able to fold with NPII and thus also to form the disulfide bond between C1 and C6. The pulse-chase experiment also shows that HN10 cells inefficiently process the precursors, since the majority of wild-type and V5xA was released as full-length protein still containing the glycopeptide. No processed precursor even of the wild-type protein was detected within the cells after the chase, suggesting that storage in granules is low in HN10 cells.

### The vasopressin and glycopeptide segments also mediate sorting into secretory granules

**Fig. 7:**
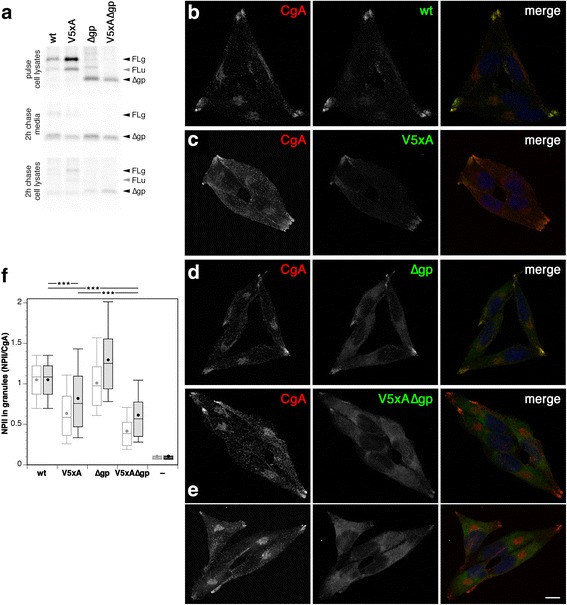
Granule sorting is mediated by both vasopressin and glycopeptide. **a** Stable AtT20 cells expressing the indicated provasopressin constructs were labeled with [^35^S]methionine for 30 min and chased for an additional 2 h. From cell lysates and media, products were immunoprecipitated and analyzed by gel electrophoresis and autoradiography. The positions of full-size glycosylated (*FLg*) and unglycosylated forms (*FLu*) and of constructs without glycopeptide (∆*gp*) are indicated. **b**–**e** AtT20 cells stably expressing wild-type provasopressin (*wt*) or the mutants without the glycopeptide (∆*gp*) or with the V5xA mutant hormone sequence (*5xA*) or both (*5xA∆gp*) were stained for CgA as a granule marker, NPII, and nuclei (*blue*). Granules concentrate in terminal patches. *Bar*: 10 μm. **f** Quantitation of NPII staining in granule patches normalized for content of CgA. NPII/CgA ratios of ~70 images per construct (45 images for untransfected AtT20 cells, *−*) containing 4–5 cells each are summarized as a *box plot* showing the median and the center 50% of values in the *box*, with the *whiskers* including the 10th to the 90th percentiles. The *central dot* represents the mean. The original quantitation is shown on the *left* in *gray* and *white*. In *black* and *gray*, the data are shown upon correction for the relative amounts of pulse-labeled protein recovered from both media and cell lysates after a 2-h chase (three independent experiments including that shown in panel **a**; wt, 1.0; V5xA, 0.77 ± 0.20; ∆gp, 0.78 ± 0.20; V5xA∆gp, 0.68 ± 0.19). All pairwise comparisons, except wild-type vs. V∆gp, are significantly different according to an unpaired two-tailed *t* test (*p* < 0.001). The most important ones are indicated above the graph

To investigate granule sorting of wild-type provasopressin and the V5xA, ∆gp, and double mutants in a quantitative manner, we generated stable expressing cells in the mouse pituitary corticotropic cell line AtT20, which is frequently used to study regulated prohormone secretion. Pulse labeling with [^35^S]methionine/cysteine for 30 min showed similar expression levels of the different constructs except for V5xA, which was produced at almost twice the amount (Fig. 7a). After a subsequent 2 h-chase, however, similar amounts of processed NPII were secreted from all cell pools, even of V5xA, suggesting that a fraction of this protein was retained in the ER. Consistent with this interpretation, unprocessed full-length forms of V5xA were still detectable in the cell lysate. While V5xA is capable of precursor folding and ER exit, it appears to be less efficient than for wild-type vasopressin in AtT20 cells.

By immunofluorescence microscopy using an NPII-specific antibody, wild-type provasopressin was found clearly concentrated in granules in the cellular extensions together with endogenous CgA as a granule marker (Fig. 7b). Separate 5xA mutation of vasopressin (Fig. 7c) or deletion of the glycopeptide (Fig. 7d) did not abolish apparent concentration in granules. In contrast, the double mutant V5xA∆gp was clearly reduced and frequently almost absent in granules (Fig. 7e).

Quantitation of provasopressin intensity in CgA-positive granules normalized to CgA content (Fig. 7f, gray) confirmed that deletion of the glycopeptide alone did not notably diminish accumulation in granules. Mutation of vasopressin, however, produced a significant reduction that was further enhanced in the double mutant. This suggests that both vasopressin and the glycopeptide contribute to sorting into granules, which is most strongly reduced when both domains are inactivated. For a more quantitative assessment of these contributions, the fluorescence values should be normalized to the levels of each protein reaching the TGN. Because ER exit of V5xA and probably also V5xA∆gp is less efficient than that of wild-type precursor and ∆gp, respectively (Fig. 7a), the levels of protein synthesis are not useful. Instead, we used the relative amounts of protein recovered after 2 h of chase — secreted into the medium and still present within the cells — when most ER-retained material had been degraded, as a better approximation to the amount of protein that exited the ER and passed through the TGN in the four cell lines. The result of this normalization is shown in Fig. 7f (black) and confirms the conclusion that both vasopressin and glycopeptide contribute to granule sorting.

### Stimulated secretion depends on vasopressin and glycopeptide

**Fig. 8:**
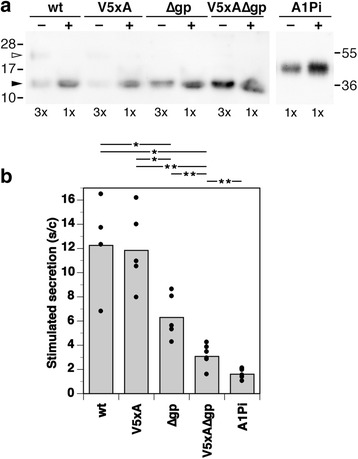
Stimulated secretion depends on both vasopressin and glycopeptide. **a** AtT20 cells stably expressing wild-type provasopressin (*wt*) or the mutants without the glycopeptide (∆*gp*) or with the V5xA mutant hormone sequence (*5xA*) or both (*5xA∆gp*), or expressing the constitutive secretory protein A1Pi were grown and incubated for 30 min each with serum-free medium without (−) and then with BaCl_2_ (+) to stimulate granule secretion for 30 min each. The media were collected, concentrated, and analyzed by SDS-gel electrophoresis and immunoblotting. To keep intensities within the diagnostic range of immunoblot analysis, a threefold larger aliquot (3x) of the control medium was analyzed than of the stimulation medium (1x). **b** Immunoblots as in A were quantified and stimulated secretion plotted as the ratio of stimulated to constitutive secretion (*s/c*). The mean and the individual values of four to five independent experiments are shown. Except for wild-type vs. V5xA, all pairwise comparisons are significantly different according to an unpaired two-tailed *t* test (the most important ones are indicated above the graph; *p < 0.05; **p < 0.01)

As a functional test for sorting into the regulated secretory pathway, we assessed the stimulated release of wild-type and mutant provasopressins from the stable AtT20 cell lines. This approach is largely independent of protein levels at the TGN. Proteins released from the different AtT20 cell lines were collected from the media before and after stimulation with BaCl_2_, corresponding to constitutive and stimulated secretion, respectively [19, 20], and analyzed by gel electrophoresis and immunoblotting. Release of wild-type and V5xA precursors were similarly stimulated more than tenfold above constitutive levels, whereas the effect of stimulation on secretion of the precusors lacking the glycopeptide was strongly reduced, most significantly for the double mutant V5xA∆gp (Fig. 8). As a negative control, stable transfected AtT20 cells expressing α1-protease inhibitor (A1Pi), a constitutive secretory protein, were analyzed. Stimulated secretion was even lower than for the double mutant, consistent with the notion that V5xA mutation still retains residual aggregation activity. Taken together the results show that both sequences causing ER aggregation of folding-deficient provasopressin mutants are also responsible for granule sorting of the wild-type protein. This supports the notion that pathological ER aggregation of DI mutants is the result of sequences that evolved to mediate granule sorting.

## Discussion

Peptides or proteins that form amyloid aggregates in vivo have long been associated exclusively with disease. In recent years, however, an increasing number of proteins have been discovered to produce functional amyloids for physiological purposes [21, 22], such as *Escherichia coli* curli for biofilm formation [23] or human Pmel17 to organize melanin in melanosomes [24]. Maji et al. [16] provided evidence suggesting that peptide hormones are concentrated in secretory granules as functional amyloids. In circumstantial support for this notion, it has previously been noted that polypeptide hormones are over-represented among the proteins known to form amyloid fibers in human disease [25], including for example amylin (islet amyloid polypeptide), calcitonin, and atrial natriuretic factor. Provasopressin also belongs to this group, since DI mutants produce fibrillar aggregates in the ER [15] and cause cell degeneration in vivo [10–12]. This correlation suggested that sequences that have evolved for physiological aggregation in granule biogenesis may be responsible for pathological aggregations under certain conditions. We have tested this concept in the case of provasopressin by identifying the sequences causing ER aggregation of misfolding mutants and testing their contribution to granule sorting and regulated secretion.

Our search for the segments in provasopressin that cause fibrillar ER aggregation surprisingly identified two separate and independent sequences: the N-terminal vasopressin nonapeptide and the C-terminal glycopeptide. Several nonsense DI mutants lack the glycopeptide and thus aggregate only via vasopressin, indicating that this type of aggregation can cause dominant disease. Whether aggregation via the glycopeptide alone is cytotoxic in vivo is not known. Three dominant DI mutations altering the vasopressin sequence have been identified [26–28], but it is unknown whether they abolish the ability of the hormone sequence to aggregate in the ER.

The large variety of more than 70 different known ADNDI mutations throughout the sequence of vasopressin and NPII strongly suggests that the common mechanism to cause disease is to prevent native folding of the precursor, which results in retention and accumulation in the ER. At the same time, the vasopressin sequence remains exposed and is able to form fibrillar aggregates that are further crosslinked by intermolecular oxidation of the large number of cysteines in NPII. In the absence of the hormone segment, aggregation still occurs via the glycopeptide. We observed previously that provasopressin with all its 16 cysteines mutated to serines was unable to aggregate [15]. One can thus conclude that under ER conditions aggregation by the glycopeptide requires stabilization by intermolecular disulfide crosslinks. Consistent with this general mechanism, no DI mutations have been found within the sequence of the glycopeptide, since they would not affect folding of vasopressin–NPII. The four different DI mutations in the signal peptide were shown to inhibit signal cleavage (see, e.g., [13]). As a result, the uncleaved signal again prevents protein folding, which requires a free N-terminus of vasopressin to insert into a pocket in NPII.

Most importantly, the same two precursor segments were also found to mediate granule sorting at the TGN of endocrine cells. This finding supports the hypothesis that the two aggregation processes in the ER and the TGN are related. In the wild-type situation, the precursor rapidly folds in the ER, whereby the vasopressin segment is concealed in its binding pocket in NPII, thus preventing premature aggregation. The glycopeptide, on the other hand, is unable to aggregate under ER conditions without stabilizing crosslinks. Upon ER exit and transport through the Golgi, the specific conditions in the TGN promote aggregation. These include reduced pH [29], high concentrations of Ca^2+^ and possibly other divalent cations such as Zn^2+^ and Mg^2+^ [30, 31], and the presence of glycosaminoglycans [32]. These conditions may induce a conformational change in the precursor to release vasopressin from its binding site on NPII. In vitro, however, the binding affinity of vasopressin to NPII was not reduced upon lowering the pH to 6 alone [33]. Alternatively, facilitated aggregation of the hormone may simply sequester it from the equilibrium between bound and free states of vasopressin into the polymers. Similarly, the TGN conditions are likely to enhance the aggregating properties of the glycopeptide, making its polymerization independent of stabilizing crosslinks.

The original proposal by Maji et al. [16] that granules constitute functional amyloids appears to conflict with the notion of granules forming by aggregation of folded proteins, since some free peptide sequence is required to form the amyloid. In the case of provasopressin, this is made possible by the glycopeptide, which is not part of the main fold of vasopressin with NPII, and by the fact that vasopressin may dissociate from NPII while remaining tethered by the connecting loop containing the convertase cleavage site. Pro-oxytocin is very closely related to provasopressin (74% identity in the hormone–NP sequence) except that it lacks a glycopeptide and is thus very likely to aggregate in granule biogenesis similarly via the hormone segment. Whether other prohormones also employ peptide segments that are conditionally releasable from a globular fold under TGN conditions remains to be investigated. Alternatively, unfolding of a globular protein may be required to form amyloid aggregates, as has been proposed by Jacob et al. [34] for growth hormone.

So far the evidence for the amyloid-like nature of secretory granules is mainly based on in vitro aggregation experiments with peptide hormones. Evidence at the cellular or tissue level is so far limited to staining of pituitary granules with an amyloid-specific antibody, with thioflavin T and Congo red, and to X-ray diffraction patterns of purified granules consistent with the presence of cross-β structure [16, 34, 35]. The fibrillar appearance of the ER aggregates of provasopressin mutants by EM suggests amyloid-like aggregation. Secretory granules, in contrast, do not reveal such fibrillar morphology, but display a homogeneous appearance [36], most likely because of denser packing of its cargo compared to the fibers of unfolded, chaperone-bound polypeptides in the ER.

Amyloids in a strict sense are cross-β-sheet polymers. Because of the high curvature of its backbone, the cyclic vasopressin peptide with its C1–C6 disulfide bond is unable to form a typical β-sheet. This may also explain why we were unable to stain ER aggregates with thioflavin S or the amyloid-specific antibody OC. One might speculate that an “imperfect amyloid” may be more rapidly redissolved upon stimulated secretion. (In contrast, the resistance of ER aggregates to complete disassembly is most likely due to the extensive disulfide crosslinking by cysteines of the misfolded NPII sequence.) Along the same line, it was shown for somatostatin-14, another circular peptide hormone with a disulfide bond between C3 and C14, that redissolution of in vitro fibrillar aggregates was accelerated for the oxidized circular form in comparison to that of the reduced linear form [35]. Chromogranins A and B, widespread secretory granule cargos, also contain a disulfide-linked ring, albeit of 22 residues, in their N-terminal domain that was shown to be required for granule sorting [37, 38]. Similarly, a 13-residue disulfide ring was identified as a granule determinant of pro-opiomelanocortin [39]. Disulfide rings may thus constitute a more general structural device for aggregation in the regulated secretory pathway.

## Conclusions

Here we identified two sequences in the vasopressin precursor protein to independently cause fibrillar, amyloid-like aggregation of folding-deficient mutants in the ER: the N-terminal hormone sequence and the C-terminal glycopeptide. They thus constitute the molecular basis of the aggregates leading to autophagy-associated cell death in dominant neurohypophyseal diabetes insipidus. Our finding that the same amyloidogenic sequences that cause pathogenic ER aggregation also confer granule sorting and regulated secretion in AtT20 cells provides biological support for the notion that secretory granules are functional amyloids of peptide hormones.

## Methods

### Plasmids and constructs

The cDNAs of the human wild-type vasopressin precursor and the mutants ∆E47 and a construct in which all cysteines were replaced by serines have been described before [13–15]. All other mutants were prepared by polymerase chain reaction (PCR) using mutagenic primers. For the Pro/Gly scan, the sequence coding for PGPGPGTPGP was generated at different positions by PCR amplification of 5′ portions of the provasopressin coding sequence using primers that added the 3′ extension CCAGGACCTGGACCTGGTACCGCG with an *Asp* 718 site (underlined) and of 3′ portions adding the 5′ extension GCGTGTACACCAGGTCCA with a *Bsr*GI site. Matching products were cut with *Asp* 718 and *Bsr*GI, respectively, mixed, ligated by their compatible ends, and amplified using the outside primers. Products of the size of the correct fusion were isolated and cloned into the expression plasmid. For construct Pro1, the first two codons were swapped to encode the sequence GPPGPGTPGP and to preserve signal cleavage. Codons 101–218 of GST from the plasmid pGEX-4T-2 (GE Healthcare) C-terminally tagged with a myc epitope were used as a reporter sequence and fused downstream of codons 1–75/50/25/16/10 of provasopressin and of 1–75Pro1. All cDNAs were subcloned into the pRc/RSV expression plasmid (Invitrogen) and confirmed by sequencing.

### Cell culture and transfection

The mouse hippocampal neuroblastoma cell line HN10 [40], which had been successfully used to study huntingtin aggregation [41], was grown in Dulbeccco’s modified Eagle’s medium (DMEM) containing 4500 mg/l glucose, supplemented with 10% fetal calf serum, 100 units/ml penicillin, 100 μg/ml streptomycin, and 2 mM l-glutamine at 37 °C in 5% CO_2_. Cells were transfected using Fugene HD (Promega). To induce differentiation, cells were cultured from 1 day after transfection in serum-free DMEM containing B27 supplements and 6 μM retinoic acid.

AtT20 cells were grown in DMEM containing 1000 mg/l glucose, supplemented with 10% fetal calf serum, 100 units/ml penicillin, 100 μg/ml streptomycin, 2 mM l-glutamine at 37 °C in 7.5% CO_2_. Cells were transfected with pRc/RSV encoding wild-type or mutant preprovasopressin or pre-A1Pi using Fugene HD and selected using 100 mg/ml G418 to select stably expressing mixed cell pools.

### Immunofluorescence

Transfected cells were grown for 48 h on glass coverslips, fixed with 3% paraformaldehyde for 30 min at room temperature, washed in phosphate-buffered saline (PBS), quenched 5 min in 50 mM NH_4_Cl in PBS, permeabilized in 0.1% Triton X-100 (Applichem) in PBS for 10 min, blocked with 1% bovine serum albumin (BSA, Roche) in PBS for 15 min, incubated at room temperature with primary antibodies for 2 h in BSA/PBS, washed, stained with fluorescent secondary antibodies in BSA/PBS for 30 min, and mounted in Fluoromount-G (Hoechst) with 0.5 μg/ml DAPI. As primary antibodies, we used a self-made polyclonal rabbit anti-provasopressin antiserum (raised against residues 28–145, i.e., the C-terminal portion of NPII and the glycopeptide), mouse monoclonal anti-myc antibody 9E10 (prepared from hybridoma, RRID:CVCL_L708, 1:100), mouse monoclonal anti-His_6_ (HIS.H8, Millipore, 05–949, Lot: 2426469, RRID:AB_492660, 1:2000), goat polyclonal anti-CgA (Santa Cruz, Sc-1488, Lot: H310, RRID:AB_2276319, 1:50), rabbit anti-A1Pi (Neomarkers, RB-367-A, Lot: 367A808B, RRID:AB_59584), and a mouse monoclonal anti-KDEL antibody (MBL, SR-827F, Lot: 12031335, RRID:AB_1279100, 1:500). As secondary antibodies, non-cross-reacting A488-labeled donkey anti-mouse (Molecular Probes, A21202, Lot: 1305303, RRID:AB_141607, 1:400), A488-labeled donkey anti-rabbit (Molecular Probes, A21206, Lot: 1674651, RRID:AB_2535792, 1:400), A568-labeled donkey anti-goat (Molecular Probes, A11057, Lot: 1235787, RRID:AB_142581, 1:400), and A568-goat anti-mouse immunoglobulin antibodies (Molecular Probes, A11031, RRID:AB_144696, 1:400) were used. Staining patterns were analyzed using a Zeiss Confocal LSM700 microscope.

Aggregation was quantified by counting the fraction of expressing cells containing punctate accumulations using a Zeiss Axioplan microscope with a Leica DFC420C imaging system. For each construct, ~200 expressing cells were counted in each of three independent transfections.

To quantify localization of provasopressin constructs with and without the glycopeptide to secretory granules, the anti-provasopressin antiserum was depleted of antibodies directed against the glycopeptide. For this, a GST-glycopeptide fusion protein was expressed in *Escherichia coli* Rosetta using the vector pGEX-4T-2 and purified with glutathione Sepharose-4B (GE Healthcare) according to the manufacturer’s instructions. Anti-provasopressin-antiserum was incubated with GST-glycopeptide bound to glutathione Sepharose-4B, and the supernatant containing only anti-NPII antibodies was tested for lack of reactivity with GST-glycopeptide by immunoblotting.

### Immunoblot analysis

Transiently transfected HN10 cells were scraped and boiled in SDS sample buffer and stored frozen. Samples were supplemented with or without 100 mM DTT, boiled, separated on 10% polyacrylamide Tris/tricine SDS-gel electrophoresis, and subjected to immunoblot analysis on polyvinylidene difluoride (PVDF) membranes (Millipore) using peroxidase-coupled secondary antibodies and the ECL (enhanced chemiluminescence) reaction kit (Millipore).

### Pulse-chase and immunoprecipitation

Transiently transfected HN10 cells or pooled stable AtT20 cells were incubated for 30 min in starvation medium (DMEM lacking methionine and cysteine) and for 30 min in labeling medium containing 100 μCi/ml [^35^S]methionine/cysteine (Hartmann Analytics, Braunschweig, Germany). For the chase, cells were incubated for an additional 2 h in complete DMEM. Cells were lysed in PBS, 1% Triton X-100, 0.5% deoxycholate, and 2 mM phenylmethylsulfonyl fluoride for 1 h at 4 °C, scraped, and centrifuged for 10 min in a microfuge. Media were collected and centrifuged for 15 min at 3000 × g. Supernatants were supplemented with an equal volume of 2X lysis buffer. Immunoprecipitation was performed using the anti-NPII antiserum and protein A Sepharose. Samples were boiled in SDS sample buffer containing 100 mM DTT and analyzed by 10% polyacrylamide Tris/tricine SDS-gel electrophoresis and autoradiography using a phosphorimager.

### Stimulated secretion

AtT20 cells stably expressing wild-type or mutant provasopressin or A1Pi were grown in 10-cm dishes, washed with PBS, and then incubated for 30 min in 3 ml secretion medium (Earle’s balanced salt solution without Ca^2+^ and Mg^2+^, supplemented with MEM amino acids, 2 mM l-glutamine, MgSO_4_, and 2 mM CaCl_2_; Sigma). After collecting the media, the cells were washed with PBS and incubated for another 30 min in stimulation medium (secretion medium without CaCl_2_, but containing 2 mM BaCl_2_). Collected secretion and stimulation media were centrifuged for 15 min at 3000 × g, and the supernatants were concentrated using Amicon Ultra-2 (molecular weight cut-off 3 kDa; Millipore). Samples were analyzed by immunoblotting.

### Electron microscopy

Cultured cells were fixed in 3% formaldehyde and 0.2% glutaraldehyde for 2 h at room temperature. Cells were then scraped, pelleted, resuspended, and washed three times in PBS, incubated with 50 mM NH_4_Cl in PBS for 30 min, washed three times in PBS, resuspended in 2% warm agarose, and left to solidify on ice. Agarose pieces were dehydrated and infiltrated with LR gold resin (London Resin, London, UK) and allowed to polymerize for 1 day at −10 °C. For immunogold labeling, sections of 60–70 nm were collected on carbon-coated Formvar-Ni-grids, incubated with our rabbit anti-provasopressin, with a rabbit anti-myc antibody (Abcam, Ab-9106, RRID:AB_307014) or with rabbit anti-calreticulin antibody (Stressgen, SPA-600, Lot: 004417, RRID:AB_10618853) in PBS, 2% BSA, 0.1% Tween-20 for 2 h, washed with PBS, and incubated with 10-nm colloidal gold-conjugated goat anti-rabbit immunoglobulin antibodies (BB International, EM GAR10, RRID:AB_1769128, 1:100) in PBS, 2% BSA, and 0.1% Tween-20 for 90 min. Grids were washed five times for 5 min in PBS and then five times in H_2_O, before staining for 10 min in 2% uranyl acetate. Sections were viewed with a Phillips CM100 electron microscope.

## Additional files

Additional file 1:
**Figure S1.**. Localization of wild-type provasopressin and the ∆E47 DI mutant in transfected HN10 cells. Immunofluorescence localization of wild-type (*wt*) provasopressin and the DI mutant precursor ∆E47 expressed in HN10 cells 48 h after transfection. Cells were costained with antibodies against provasopressin (*V*; *blue*), KDEL as an ER marker (*red*), and CgA as a cargo of secretory granules (*green*). Wild-type provasopressin is detected in granules in the neuronal extension together with CgA (*filled arrowheads*), whereas ∆E47 in a majority of expressing cells is found in aggregates in the ER of the cell body costained with anti-KDEL (*open arrowheads*). *Bar*: 10 μm. (TIF 1759 kb)
Additional file 2:
**Figure S2.**. The second aggregation motif also causes fibrillar aggregation. **A**–**D** Electron microscopy of Pro1 (A and B) or ∆E47Pro1 (C and D) decorated with immunogold for provasopressin (A, C, and D) or for calreticulin (B). The ∆E47 point mutation made no observable difference in the range of aggregate morphologies. *Bars*: 500 nm. (TIF 9565 kb)
Additional file 3:
**Figure S3.**. Vasopressin residues binding into NPII. NPII (*blue*) with bound vasopressin (*green*) is shown according to the crystal structure 1JK4 [1]. Residues P7, R8, and G9, which were not resolved in the structure, are presented in an arbitrary conformation. On the right, the portion of vasopressin interacting with NPII is shown in *blue*. (TIF 520 kb)

## References

[1] Wu CK, Hu B, Rose JP, Liu ZJ, Nguyen TL, Zheng C (2001). Structures of an unliganded neurophysin and its vasopressin complex: implications for binding and allosteric mechanisms. Protein Sci.

[2] De Bree FM, Van Der Kleij AAM, Nijenhuis M, Zalm R, Murphy D, Burbach JPH (2003). The hormone domain of the vasopressin prohormone is required for the correct prohormone trafficking through the secretory pathway. J Neuroendocrinol.

[3] Arvan P, Castle D (1998). Sorting and storage during secretory granule biogenesis: looking backward and looking forward. Biochem J.

[4] Kim T, Gondré-Lewis MC, Arnaoutova I, Loh YP (2006). Dense-core secretory granule biogenesis. Physiology (Bethesda).

[5] Dikeakos JD, Reudelhuber TL (2007). Sending proteins to dense core secretory granules: still a lot to sort out. J Cell Biol.

[6] Dannies PS (2012). Prolactin and growth hormone aggregates in secretory granules: the need to understand the structure of the aggregate. Endocr Rev.

[7] Beuret N, Stettler H, Renold A, Rutishauser J, Spiess M (2004). Expression of regulated secretory proteins is sufficient to generate granule-like structures in constitutively secreting cells. J Biol Chem.

[8] Green JR, Buchan GC, Alvord GC, Swanson AG (1967). Hereditary and idiopathic types of diabetes insipidus. Brain.

[9] Nagai I, Li CH, Hsieh SM, Kizaki T, Urano Y (1984). Two cases of hereditary diabetes insipidus, with an autopsy finding in one. Acta Endocrinol (Copenh).

[10] Bergeron C, Kovacs K, Ezrin C, Mizzen C (1991). Hereditary diabetes insipidus: an immunohistochemical study of the hypothalamus and pituitary gland. Acta Neuropathol.

[11] Russell TA, Ito M, Ito M, Yu RN, Martinson FA, Weiss J (2003). A murine model of autosomal dominant neurohypophyseal diabetes insipidus reveals progressive loss of vasopressin-producing neurons. J Clin Invest.

[12] Hagiwara D, Arima H, Morishita Y, Wenjun L, Azuma Y, Ito Y (2014). Arginine vasopressin neuronal loss results from autophagy-associated cell death in a mouse model for familial neurohypophysial diabetes insipidus. Cell Death Dis.

[13] Beuret N, Rutishauser J, Bider MD, Spiess M (1999). Mechanism of endoplasmic reticulum retention of mutant vasopressin precursor caused by a signal peptide truncation associated with diabetes insipidus. J Biol Chem.

[14] Friberg MA, Spiess M, Rutishauser J (2004). Degradation of wild-type vasopressin precursor and pathogenic mutants by the proteasome. J Biol Chem.

[15] Birk J, Friberg MA, Prescianotto-Baschong C, Spiess M, Rutishauser J (2009). Dominant pro-vasopressin mutants that cause diabetes insipidus form disulfide-linked fibrillar aggregates in the endoplasmic reticulum. J Cell Sci.

[16] Maji SK, Perrin MH, Sawaya MR, Jessberger S, Vadodaria K, Rissman RA (2009). Functional amyloids as natural storage of peptide hormones in pituitary secretory granules. Science.

[17] De Bree FM, Knight D, Howell L, Murphy D (2000). Sorting of the vasopressin prohormone into the regulated secretory pathway. FEBS Lett.

[18] Cool D, Jackson S, Waddell K (2008). Structural requirements for sorting pro-vasopressin to the regulated secretory pathway in a neuronal cell line. Open Neuroendocrinol J.

[19] Zhu YL, Conway-Campbell B, Waters MJ, Dannies PS (2002). Prolonged retention after aggregation into secretory granules of human R183H-growth hormone (GH), a mutant that causes autosomal dominant GH deficiency type II. Endocrinology.

[20] Bonnemaison M, Bäck N, Lin Y, Bonifacino JS, Mains R, Eipper B (2014). AP-1A controls secretory granule biogenesis and trafficking of membrane secretory granule proteins. Traffic.

[21] Chiti F, Dobson CM (2006). Protein misfolding, functional amyloid, and human disease. Annu Rev Biochem.

[22] Fowler DM, Koulov AV, Balch WE, Kelly JW (2007). Functional amyloid — from bacteria to humans. Trends Biochem Sci.

[23] Blanco LP, Evans ML, Smith DR, Badtke MP, Chapman MR (2012). Diversity, biogenesis and function of microbial amyloids. Trends Microbiol.

[24] Fowler DM, Koulov AV, Alory-Jost C, Marks MS, Balch WE, Kelly JW (2006). Functional amyloid formation within mammalian tissue. PLoS Biol.

[25] Westermark GT, Sipe JD (2005). Endocrine amyloid. Amyloid proteins: the beta sheet conformation and disease.

[26] Rittig S, Siggaard C, Ozata M, Yetkin I, Gregersen N, Pedersen EB (2002). Autosomal dominant neurohypophyseal diabetes insipidus due to substitution of histidine for tyrosine(2) in the vasopressin moiety of the hormone precursor. J Clin Endocrin Metab.

[27] Kobayashi H, Fujisawa I, Ikeda K, Son C, Iwakura T, Yoshimoto A (2006). A novel heterozygous missense mutation in the vasopressin moiety is identified in a Japanese person with neurohypophyseal diabetes insipidus. J Endocrinol Invest.

[28] Wahlstrom JT, Fowler MJ, Nicholson WE, Kovacs WJ (2004). A novel mutation in the preprovasopressin gene identified in a kindred with autosomal dominant neurohypophyseal diabetes insipidus. J Clin Endocrin Metab.

[29] Paroutis P, Touret N, Grinstein S (2004). The pH of the secretory pathway: measurement, determinants, and regulation. Physiology (Bethesda).

[30] Thorlacius-Ussing O (1987). Zinc in the anterior pituitary of rat: a histochemical and analytical work. Neuroendocrinology.

[31] Hutton JC, Penn EJ, Peshavaria M (1983). Low-molecular-weight constituents of isolated insulin-secretory granules. Bivalent cations, adenine nucleotides and inorganic phosphate. Biochem J.

[32] Kolset SO, Prydz K, Pejler G (2004). Intracellular proteoglycans. Biochem J.

[33] De Bree FM, Burbach JP (1998). Structure-function relationships of the vasopressin prohormone domains. Cell Mol Neurobiol.

[34] Jacob RS, Das S, Ghosh S, Anoop A, Jha NN, Khan T (2016). Amyloid formation of growth hormone in presence of zinc: relevance to its storage in secretory granules. Sci Rep Nat Pub Group.

[35] Anoop A, Ranganathan S, Dhaked Das B, Jha NN, Pratihar S, Ghosh S (2014). Elucidating the role of disulfide bond on amyloid formation and fibril reversibility of somatostatin-14: relevance to its storage and secretion. J Biol Chem.

[36] Cross PC, Mercer KL (1998). Cell and tissue ultrastructure.

[37] Krömer A, Glombik MM, Huttner WB, Gerdes HH (1998). Essential role of the disulfide-bonded loop of chromogranin B for sorting to secretory granules is revealed by expression of a deletion mutant in the absence of endogenous granin synthesis. J Cell Biol.

[38] Courel M, Rodemer C, Nguyen ST, Pance A, Jackson AP, O’Connor DT (2006). Secretory granule biogenesis in sympathoadrenal cells: identification of a granulogenic determinant in the secretory prohormone chromogranin A. J Biol Chem.

[39] Cool DR, Fenger M, Snell CR, Loh YP (1995). Identification of the sorting signal motif within pro-opiomelanocortin for the regulated secretory pathway. J Biol Chem.

[40] Lee HJ, Hammond DN, Large TH, Roback JD, Sim JA, Brown DA (1990). Neuronal properties and trophic activities of immortalized hippocampal cells from embryonic and young adult mice. J Neurosci.

[41] Weiss A, Roscic A, Paganetti P (2009). Inducible mutant huntingtin expression in HN10 cells reproduces Huntington’s disease-like neuronal dysfunction. Mol Neurodegener.

[42] Conchillo-Solé O, de Groot NS, Avilés FX, Vendrell J, Daura X, Ventura S (2007). AGGRESCAN: a server for the prediction and evaluation of “hot spots” of aggregation in polypeptides. BMC Bioinformatics.

[43] Frousios KK, Iconomidou VA, Karletidi C-M, Hamodrakas SJ (2009). Amyloidogenic determinants are usually not buried. BMC Struct Biol.

[44] Garbuzynskiy SO, Lobanov MY, Galzitskaya OV (2010). FoldAmyloid: a method of prediction of amyloidogenic regions from protein sequence. Bioinformatics.

