# Supplementary figures and images for: Amyloid-like aggregation of provasopressin in diabetes insipidus and secretory granule sorting
Source: BMC Biol. 2017 Jan 26;15:5. doi: 10.1186/s12915-017-0347-9 (PMC5267430; doi:10.1186/s12915-017-0347-9)

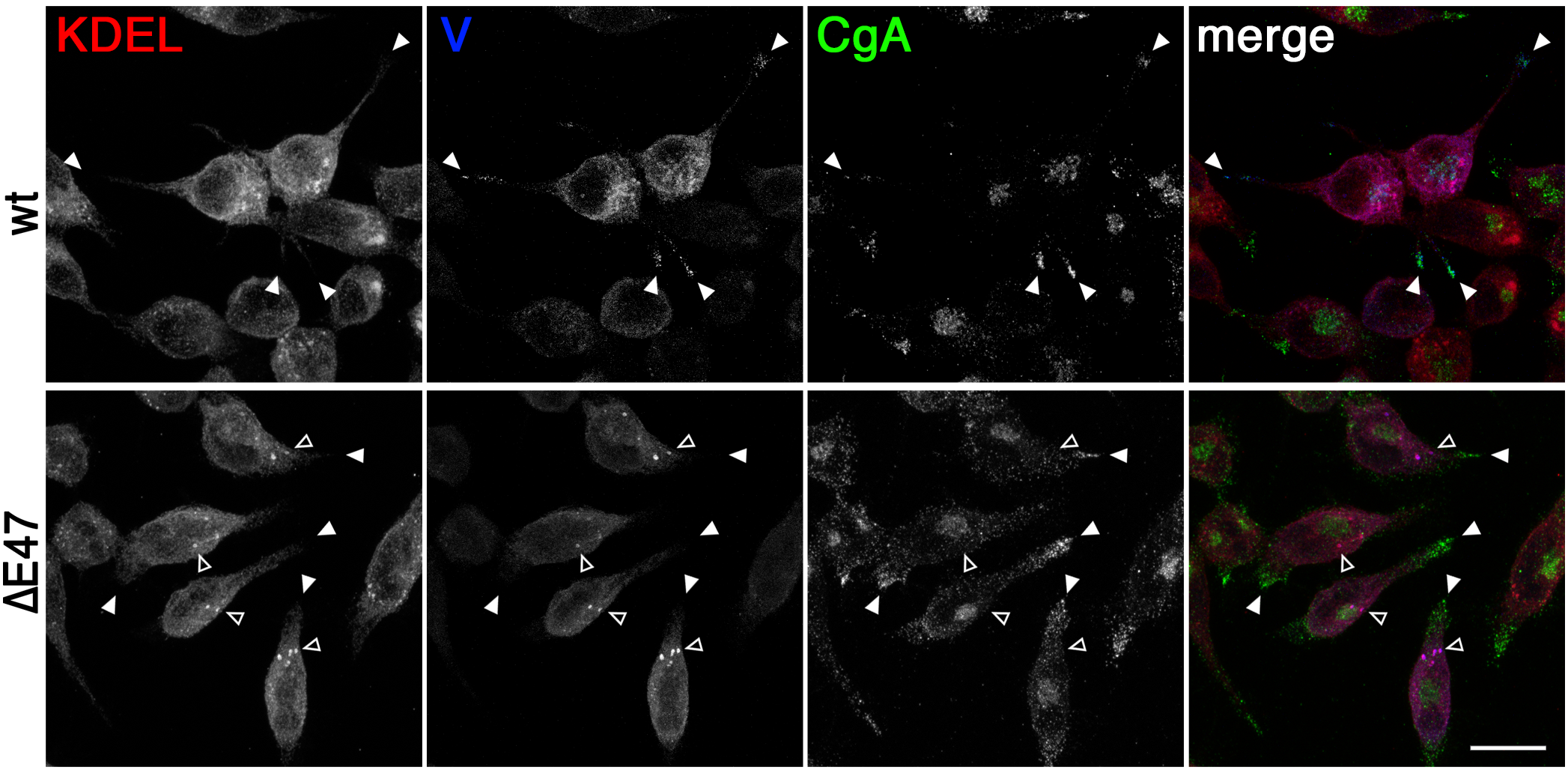

Supplement: Additional file 1: — Figure S1.. Localization of wild-type provasopressin and the ∆E47 DI mutant in transfected HN10 cells. Immunofluorescence localization of wild-type (wt) provasopressin and the DI mutant precursor ∆E47 expressed in HN10 cells 48 h after transfection. Cells were costained with antibodies against provasopressin (V; blue), KDEL as an ER marker (red), and CgA as a cargo of secretory granules (green). Wild-type provasopressin is detected in granules in the neuronal extension together with CgA (filled arrowheads), whereas ∆E47 in a majority of expressing cells is found in aggregates in the ER of the cell body costained with anti-KDEL (open arrowheads). Bar: 10 μm. (TIF 1759 kb) [file 12915_2017_347_MOESM1_ESM.tif]

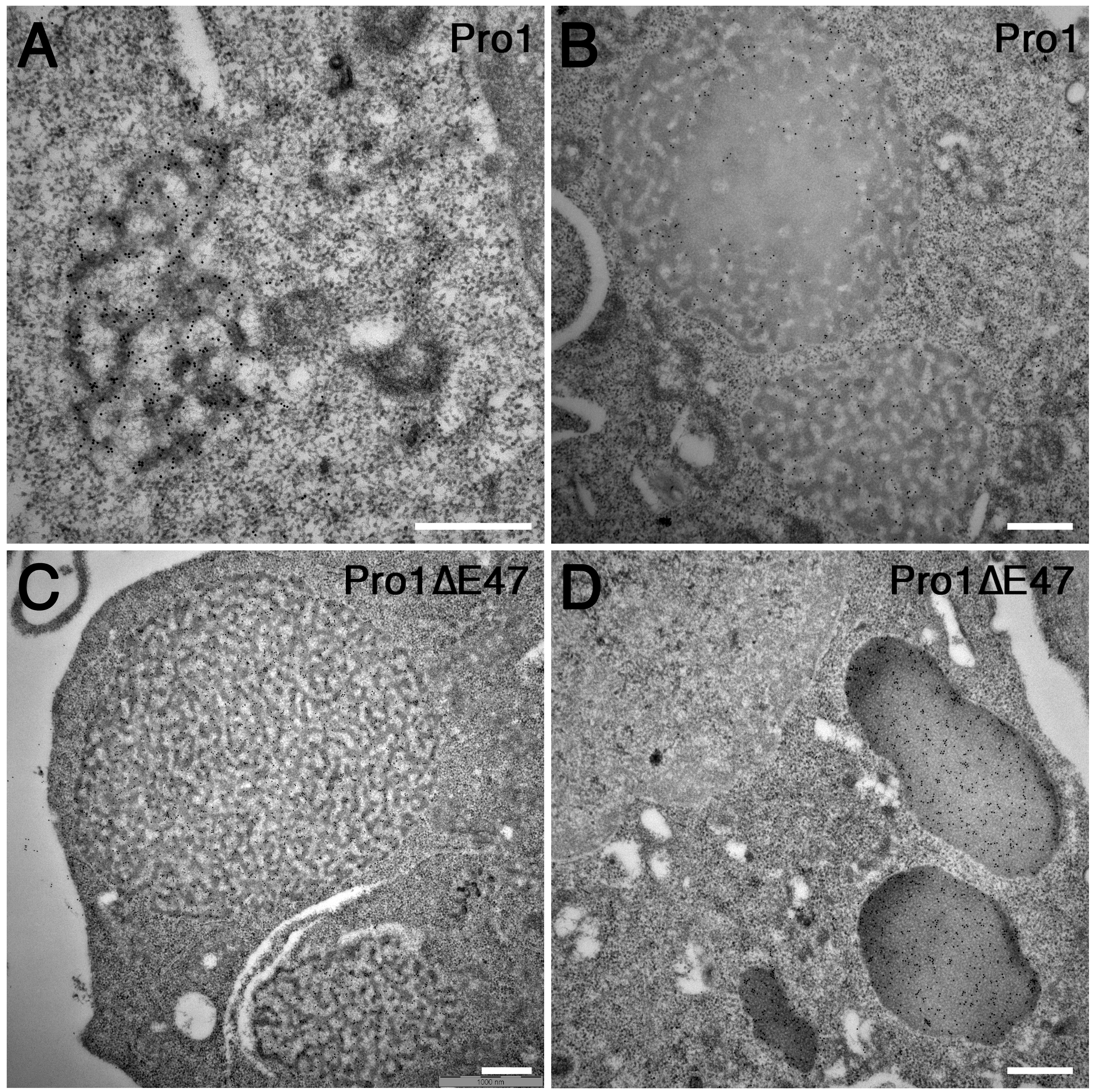

Supplement: Additional file 2: — Figure S2.. The second aggregation motif also causes fibrillar aggregation. A–D Electron microscopy of Pro1 (A and B) or ∆E47Pro1 (C and D) decorated with immunogold for provasopressin (A, C, and D) or for calreticulin (B). The ∆E47 point mutation made no observable difference in the range of aggregate morphologies. Bars: 500 nm. (TIF 9565 kb) [file 12915_2017_347_MOESM2_ESM.tif]

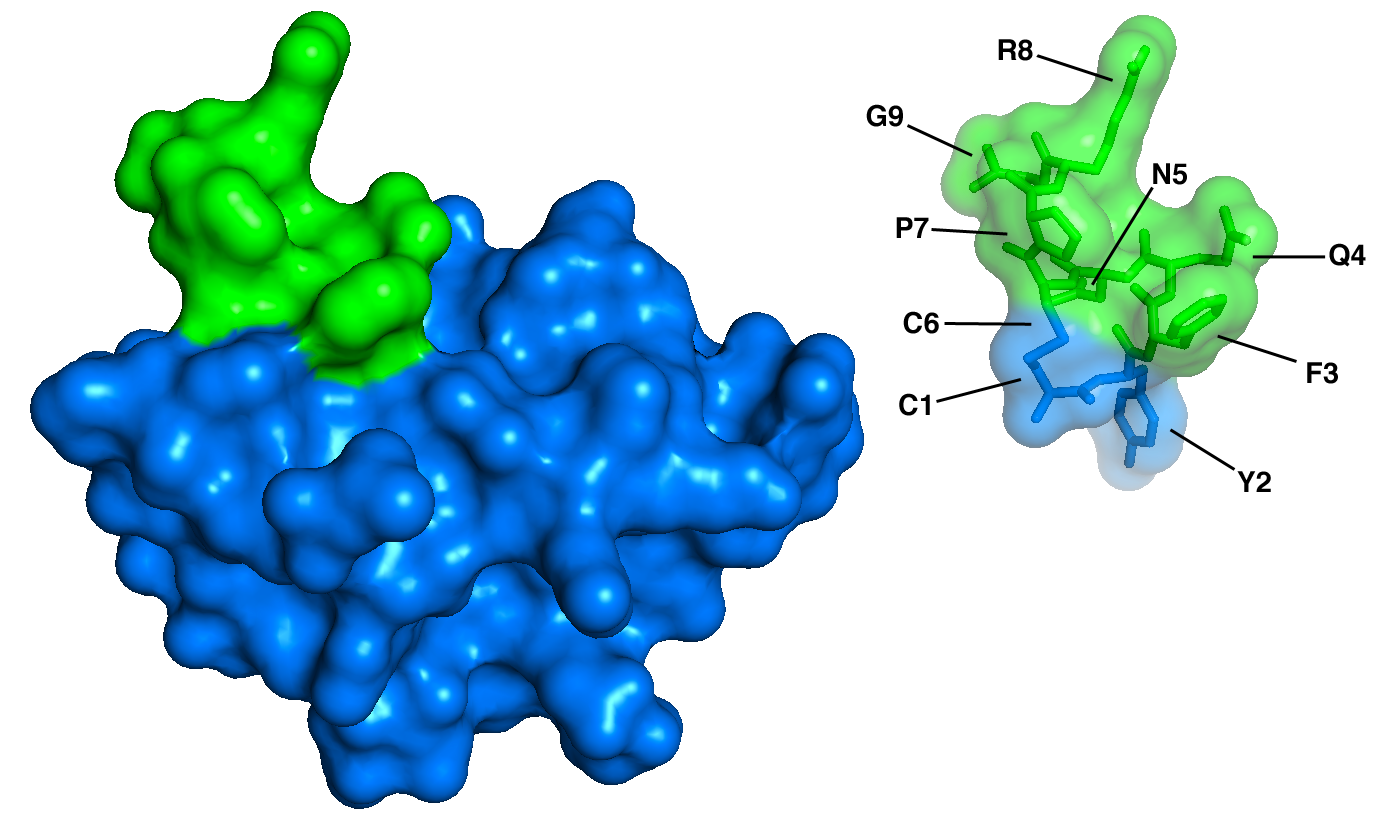

Supplement: Additional file 3: — Figure S3.. Vasopressin residues binding into NPII. NPII (blue) with bound vasopressin (green) is shown according to the crystal structure 1JK4 [1]. Residues P7, R8, and G9, which were not resolved in the structure, are presented in an arbitrary conformation. On the right, the portion of vasopressin interacting with NPII is shown in blue. (TIF 520 kb) [file 12915_2017_347_MOESM3_ESM.tif]
